# Assessment of the Antiviral Properties of Recombinant Porcine SP-D against Various Influenza A Viruses *In Vitro*

**DOI:** 10.1371/journal.pone.0025005

**Published:** 2011-09-14

**Authors:** Marine L. B. Hillaire, Martin van Eijk, Stella E. van Trierum, Debby van Riel, Xavier Saelens, Roland A. Romijn, Wieger Hemrika, Ron A. M. Fouchier, Thijs Kuiken, Albert D. M. E. Osterhaus, Henk P. Haagsman, Guus F. Rimmelzwaan

**Affiliations:** 1 Erasmus Medical Centre, Rotterdam, The Netherlands; 2 Department of Infectious Diseases and Immunology, Faculty of Veterinary Medicine, Utrecht University, Utrecht, The Netherlands; 3 Department for Molecular Biomedical Research, VIB, Ghent, Belgium; 4 Department of Biomedical Molecular Biology, Ghent University, Ghent, Belgium; 5 U-Protein Express B.V., Science Park Utrecht, Utrecht, The Netherlands; 6 Viroclinics Biosciences BV, Rotterdam, The Netherlands; Duke University School of Medicine, United States of America

## Abstract

The emergence of influenza viruses resistant to existing classes of antiviral drugs raises concern and there is a need for novel antiviral agents that could be used therapeutically or prophylacticaly. Surfactant protein D (SP-D) belongs to the family of C-type lectins which are important effector molecules of the innate immune system with activity against bacteria and viruses, including influenza viruses. In the present study we evaluated the potential of recombinant porcine SP-D as an antiviral agent against influenza A viruses (IAVs) *in vitro*. To determine the range of antiviral activity, thirty IAVs of the subtypes H1N1, H3N2 and H5N1 that originated from birds, pigs and humans were selected and tested for their sensitivity to recombinant SP-D. Using these viruses it was shown by hemagglutination inhibition assay, that recombinant porcine SP-D was more potent than recombinant human SP-D and that especially higher order oligomeric forms of SP-D had the strongest antiviral activity. Porcine SP-D was active against a broad range of IAV strains and neutralized a variety of H1N1 and H3N2 IAVs, including 2009 pandemic H1N1 viruses. Using tissue sections of ferret and human trachea, we demonstrated that recombinant porcine SP-D prevented attachment of human seasonal H1N1 and H3N2 virus to receptors on epithelial cells of the upper respiratory tract. It was concluded that recombinant porcine SP-D holds promise as a novel antiviral agent against influenza and further development and evaluation in vivo seems warranted.

## Introduction

IAV are a major cause of respiratory tract infections and cause excess morbidity and mortality every year. In addition to seasonal outbreaks of influenza epidemics in the winter months, novel influenza viruses are occasionally introduced into the human population and cause pandemics. The introduction of IAV of the H1N1, H2N2 and H3N2 subtypes have caused pandemics in 1918, 1957 and 1968 respectively. More recently, H1N1 viruses of swine origin caused the first pandemic of the 21^st^ century, which started in Mexico in March 2009 [1], [2]. The virus spread rapidly over all continents and caused disease especially in children and young adults [3].

In addition, avian IAVs are occasionally transmitted from infected poultry to man. Especially highly pathogenic avian influenza viruses of the H5N1 subtype cause infections of humans relatively frequent and 60% of these cases have a fatal outcome [4], [5].

To protect individuals against seasonal IAV infection, vaccination is the preventive measure of choice. Especially individuals with high risk to develop complications of IAV infection benefit from vaccination, like the elderly, patients with chronic diseases and immunocompromised individuals. In addition, vaccination could help to mitigate the impact of a pandemic outbreak. However, there are three scenarios in which vaccination cannot be used effectively. First, certain high risk group respond to vaccination poorly like the frail elderly and certain immunocompromised subjects. Secondly, in case of antigenic mis-match, the vaccine may induce antibodies that fail to neutralize the epidemic strains optimally and therefore do not afford protection against theses drift variants [6], [7]. Finally, in case of a pandemic the vaccines may become available too late as was the case during the 2009 pandemic [2].

Under circumstances mentioned above, antiviral drugs may be used to treat patients infected with IAV and, alternatively, such drugs can be used prophylactically in outbreak situations.

At present, two classes of licensed antiviral drugs against influenza exist. The first are the adamantanes (amantadine and rimantadine) which inhibit the proton-channel function of the Matrix2 (M2) protein. This protein functions as an ion-channel and plays a role in the uncoating of the genetic material which is an essential step in the virus replication cycle.

The other class of antiviral drugs comprises the neuraminidase (NA) inhibitors, zanamivir and oseltamivir. NA acts as a receptor destroying enzyme and plays a role in the release of viral particles that bud from infected cells. NA inhibitors are analogs of sialic acid, the substrate of the enzyme. The use of these drugs reduces virus replication and may reduce the duration of influenza illness [8], [9], [10].

Unfortunately the use of these antiviral drugs also has some drawbacks. Especially the use of M2 inhibitors may cause some side effects including neurological and gastrointestinal symptoms. The main drawback of the use of both M2 and NA inhibitors is the emergence of resistant strains which are positively selected rapidly. For example, seasonal H3N2 IAV resistant to amantadine emerged in 2000 in Asia and is the predominant strain ever since [11]. Furthermore, 15.5% of the H1N1 viruses were resistant to adamantanes in 2005–2006 worldwide. In addition, most of the seasonal H1N1 viruses isolated in the influenza season 2008–2009 were resistant to oseltamivir [11], [12], [13]. Also during the treatment of especially immunocompromised patients, which shed virus for extensive periods, the emergence of resistant strains can be a complication.

Clearly there is a need for novel drugs that can be used to treat IAV infection and that do not suffer from these disadvantages.

Therefore, we wished to assess the antiviral properties of recombinant SP-D from human (RhSP-D) and porcine (RpSP-D) origin to various IAV and their potential as an antiviral drug. It is hypothesized that IAV less likely will develop resistance to this class of antiviral molecules since they target glycans present on IAV. SP-D belongs to the collectin family, which is an important class of innate immunity proteins involved in the early response against pathogens. These collagenous C-type lectins are mainly produced by Clara cells and type II pneumocytes. They can neutralize a variety of respiratory pathogens including bacteria and viruses such as respiratory syncytial virus, parainfluenza virus, adenoviruses, SARS coronavirus and IAV (for review see [14], [15]). Collectins can bind glycoconjugates expressed on the surface of these pathogens via their carbohydrate recognition domains (CRDs).

The importance of SP-D is demonstrated by studies with SP-D knock-out mice that are more susceptible to infection with IAV than SP-D competent mice [16]. Furthermore, it has been shown that the SP-D concentration increases during IAV infection and that the administration of an SP-D inhibitor during infection of mice increased the lung virus titer compared to a normal infection [17].

The favourable biological properties of SP-D in vivo prompted further investigation of this molecule as an antiviral drug. Especially porcine SP-D (pSP-D) showed promising results in vitro [18], [19], [20]. It exhibits unique structural features compared to SP-Ds from other animal species. Unlike its human counterpart, it contains an extra cysteine in the collagen domain, it is N-glycosylated with a sialic acid-rich oligosaccharide in the CRD and there is a potentially important extra loop region (SGA) present in the CRD which could play a role in the sugar-binding properties of pSP-D [19]. It was shown with pSP-D isolated from porcine bronchoalveolar lavage that pSP-D displays a higher affinity to IAV than human or rat SP-D [19]. Since only a limited number of IAVs was tested in previous studies, it still remains unknown what range of IAV strains can be neutralized by pSP-D and by hSP-D.

In the present study, we investigated the potential of RpSP-D and RhSP-D to neutralize IAVs of the H1N1, H3N2 and H5N1 subtypes.

These findings indicate that RpSP-D has broadly neutralizing activity and may be a promising candidate as an antiviral drug for the treatment of IAV.

## Materials and Methods

### Ethics Statement

The isolation of native SP-D was performed using lungs of surplus pigs that were euthanized for other purposes. The porcine lung tissue described in this manuscript was collected from animals initially used for instructing surgeons specific surgical techniques. According to the Dutch Experiments on Animals Act an ethical assessment is required for the primary aim for which experimental procedures are to be conducted on animals. An ethical assessment is not mandatory for secondary purpose use of tissues or organs collected from carcasses. The carcasses were obtained form the facilities of the Utrecht University, the Netherlands. The Medische Ethische Toetsings Commissie (METC) of the Erasmus Medical Center exempted this study from review because according to dutch law this was not required since the study subjects were not experimentally treated or asked to change their normal routine and waived the need of consent due to the fact the samples received were surplus material from surgical specimens, with patient identifiers removed and no follow-up information obtained. These material were obtained from the archive of the department of Pathology at the Eramus Medical Center in Rotterdam, the Netherlands.

### Viruses

Thirty viruses were selected based on their subtype and species of origin and included avian swine and human IAV of the H1N1, H3N2 and H5N1 subtypes. The characteristics of these strains are listed in table 1. All the viruses were propagated in Madin-Darby Canine Kidney (MDCK, ATCC CCL-34) cells as described previously [21]. The culture supernatants of the infected MDCK were clarified by low speed centrifugation, aliquoted and stored at −80°C until use. Infectious virus titers were determined as described previously [21].

**Table 1:** Influenza A viruses used in the present study.

Subtype	Classification	Strain	Abbreviated strain name
	Pandemic 2009	A/Netherlands/602/09	NL/09
		A/California/4/09	Cali/09
	Classical swine	A/swine/Shope/11/56	S/Shope/56
		A/swine/Iowa/15/30	S/Iowa//30
		A/NewJersey/8/76	NJ/76
	Avian-like swine	A/swine/Netherlands/25/80	S/NL/80
**H1N1**		A/swine/Netherlands/1/87	S/NL/1/87
	Human	A/Netherlands/364/06	NL/06
		A/Netherlands/246/08	NL/08
		A/PuertoRico/8/34	PR8
		A/USSR/90/77	USSR/77
	Avian	A/mallard/Netherlands/15/05	M/NL/15/05
		A/mallard/Netherlands/24/06	M/NL/06
		A/white fronted goose/Netherlands/1/07	WFG/NL/07
		A/common teal/Netherlands/10/00	CT/NL/00
	Swine	A/swine/Oedenrode/7C/96	S/OR/96
		A/swine/Netherlands/849/93	S/NL/93
		A/swine/Utrecht/4/85	S/Utrecht/85
		A/swine/Ukkel/1/84	S/Ukkel/84
**H3N2**	Human	A/Netherlands/348/07	NL/07
		A/Netherlands/548/05	NL//05
		A/Netherlands/312/03	NL/03
		A/Netherlands/35/93	NL/93
	Avian	A/mallard/Netherlands/19/05	M/NL/19/05
		A/mallard/Netherlands/51/08	M/NL/08
		A/mallard/Sweden/51/03	M/Sw/03
		A/mallard/Netherlands/1/07	M/NL/07
	Clade 0	A/HongKong/156/97	HongKong/97
**H5N1**	Clade 1	A/VietNam/1194/04	VietNam/04
	Clade 2.1	A/Indonesia/5/05	Indonesia/05

The three recombinant H5N1 viruses, Indonesia/5/05, VietNam/1194/04 and HongKong/156/97 were prepared as 6+2 reassortant strains by reverse genetic as described previously [22]. The basic cleavage site in the HA protein was deleted by site-directed mutagenesis.

### Native pSP-D

Native pSP-D (NpSP-D) was isolated from pig lungs as described previously [23]. For this purpose six months old surplus pigs were used that were euthanized for other purposes. In short, NpSP-D was isolated from lung lavage by affinity purification method using Mannan-sepharose beads. After elution from the beads with EDTA-containing buffer, NpSP-D was purified using gel filtration chromatography.

### Recombinant porcine and human SP-D

RpSP-D was expressed, purified and characterized as described by van Eijk et al. (manuscript in preparation) and RhSP-D was produced based upon the full-length hSP-D clone provided by Dr. E.C. Crouch (Washington University, St. Louis, USA) [24]. Briefly, SP-D sequence-containing pUPE 101.01 expression plasmids were transfected into HEK293-EBNA1 cells (ATCC CRL-10852) using the protocol previously described by Kamen et al [25]. Expression media were harvested after 5 days, a solution of CaCl_2_ was added (10 mM final concentration) and the media were affinity purified with mannan-agarose. After elution with EDTA containing buffer (5 mM Hepes, 0.9% NaCl, 5 mM EDTA, pH 7,4), the eluted SP-D was separated by size exclusion chromatography into differently assembled forms (trimers, dodecamers). Endotoxin levels were determined with the Toxinsensor LAL assay kit and ranged between 10–100 pg/µg SP-D.

### Hemagglutination Inhibition assay

Binding of SP-D to the viral HA and interference with binding of the virus to its receptor was assessed by HI. Two-fold dilutions of SP-D or peanut agglutinin (Sigma Aldrich, Schneeldorf, Germany), which was included as a negative control, were made using Dulbecco's phosphate buffered saline containing PBS containing 1 mM of CaCl_2_ and 0.5 mM of MgCl_2_, PBS-CM (Gibco, Grand Island, USA). To 100 µl of the diluted SP-D, 2 hemagglutination units (HAU) of the respective viruses diluted in PBS-CM were added. After 1 h, 25 µl of 1% turkey erythrocytes were added. The hemagglutination patterns were read after 3 h of incubation at room temperature. As a negative control, the experiment was also performed in PBS without CaCl_2_ to demonstrate the Ca^2+^-dependency of the SP-D activity.

### Assessment of binding of RpSP-D to HA

Transient calcium phosphate-mediated transfections of 293T cells were performed essentiality as previously described [26]. Briefly, 3×10^5^cells were seeded into wells of 6 well plates and after 16 hours transfected with 5μg of plasmid from which the HA or NP genes of influenza viruses A/Netherlands/26/07 (H1N1) or A/Netherlands//178/95 (H3N2). In brief, the plasmid DNA was mixed with calcium phosphate (0.25M) in a total volume of 50 μL of water. 50 μL of HBS (1.64% NaCl, 1.18% Hepes and 0.04%Na_2_PO_4_, pH = 7.12) was added dropwise and the mixture was left at room temperature for 5 minutes. The DNA mixture was transferred to 293T cells and incubated overnight at 37°C. Then, the cells were washed with PBS-CM and incubated with 2 μg of RpSP-D for one hour at 37°C. Cells incubated without RpSP-D were included as negative controls. After washing with PBS-CM, the cells were incubated with a monoclonal antibody directed to pSP-D (AbD serotec, Oxford, UK) diluted 1∶100 in PBS-CM for 30 minutes at room temperature. The cells were washed with PBS-CM and incubated with a FITC-labeled goat anti-mouse IgG antibody preparation (AbD Sigma-Aldricht) for 30 minutes at room temperature. After washing the cells were analyzed using a fluorescence microscope (Axiovert 25, Zeiss, Sliedrecht, Netherlands).

### Infection Reduction assay

To determine the neutralizing capacity of RpSP-D an infection reduction assay was used. To this end, 100 µl of various concentrations of RpSP-D or peanut agglutinin were incubated with 100 µl of the respective virus preparations containing 3000TCID_50_ diluted in PBS-CM for 1 h at room temperature. Subsequently, the mixture was transferred to MDCK cells, which were washed with PBS-CM, and incubated for 1 h at 37°C. Then, the cells were washed once with PBS-CM and twice with PBS containing 5 mM of EDTA.

The cells were incubated for 24 h at 37°C in culture medium without trypsin to prevent secondary infection of cells by IAV, and then washed once with PBS containing EDTA and subsequently trypsinized. The cell suspensions were transferred to a 96-wells V-bottom plate and washed twice with PBS containing 2% Fetal Bovine Serum (P2F). They were stained for viability using AmCyan-labeled Live/dead staining (Invitrogen, Oregon, USA). After washing with P2F, the cells were fixed with 100 µl of cytofix (BD Biosciences, San Diego, CA) according to the manufacturer recommendations. Subsequently, the cells were washed twice with cytoperm (BD Biosciences, San Diego, CA) and incubated with a monoclonal antibody specific for the viral nucleoprotein, labeled with fluorescein isothiocyanate, FITC, (DAKOCytomation, Glostrup, Denmark). After washing twice with P2F, the cells were analyzed by flow-cytometry using the DIVA software.

### Virus histochemistry

For virus histochemistry the seasonal IAVs A/Netherlands/35/05 (H1N1) and A/Netherlands/231/03 (H3N2) were used. These viruses were propagated in MDCK cells and purified by sucrose gradient density centrifugation. They were incubated in formalin for one week and then labeled with FITC (Sigma-Aldrich, Saint Louis, MO) as described previously [27]. Archival paraffin-embedded human tracheal tissue sections were obtained from the Department of Pathology, Erasmus MC. Ethics approval was not required because it concerned surplus material from surgical specimens, with patient identifiers removed and no follow-up information obtained. Archival paraffin-embedded ferret tracheal tissue sections were obtained from the Department of Virology, Erasmus MC. The assessment of virus binding to the trachea tissue was performed essentially as described previously [27]. In brief, FITC-labeled virus was incubated with the tissue sections and binding of virus was detected using a peroxidase labeled rabbit anti-FITC antibody preparation (DAKOCytomation, Glostrup, Denmark) and the signal was amplified with a tyramide signal amplification system (Perkin Elmer, Boston, MA).

Various concentrations of RpSP-D, peanut agglutinin (negative control), pokeweed (negative control) or Concanavalin A (ConA) (positive control) were incubated with 40 HAU of virus for 1 h. All dilutions were made in PBS-CM.

The tissue sections were first deparaffinized with xylene and hydrated with ethanol. Then, they were incubated with 3% H_2_O_2_ for 10 min and subsequently incubated with Tris -HCl pH 7.5 containing 0.5% blocking reagent buffer (Perkin Elmer, Boston, MA). Then the tissue sections were incubated with FITC-labelled virus for 1 h in the absence or presence of RpSP-D or one of the other lectins for 1 h. After washing the slides with PBS containing 5 mM of EDTA and 0.05% Tween they were further developed. The tissues were counterstained with haematoxylin and embedded in glycerol-gelatin (Merck, Whitehouse Station, NJ).

### Neuraminidase Inhibition assay

To exclude steric hindrance from binding to the HA, recombinant NA was used in the NA inhibition assay. Tetrameric, GCN4 stabilized, soluble recombinant NA derived from A/Victoria/3/75 (H3N2) and A/crested eagle/Belgium/01/2004 (H5N1, cDNA kindly provided by Dr. Thierry van den Berg, VAR, Brussels) virus were expressed and purified from a recombinant baculovirus expression system in Sf/9 cells, as described [28], [29]. In brief, after baculovirus infection, Sf/9 cell culture medium was harvested, passed through a 0.22 µm filter and extracted with 3/5 volumes of n-butanol. The aquous phase, containing NA, was adjusted to 5 mM KH_2_PO_4_ pH 6.6 and loaded on a hydroxyapatite column, equilibrated with 5 mM KH_2_PO_4_ pH 6.6, 4% butanol. The column was eluted using a linear gradient of 5 mM KH_2_PO_4_ pH 6.6, 4% butanol to 400 mM KH_2_PO_4_ pH 6.6, 1% butanol. NA-containing fractions were pooled and subsequently loaded on a Blue sepharose column, followed by a wash step with 50 mM MES pH 6.6, 5% glycerol, 8 mM CaCl_2_. The Bleu sepharose column was eluted with 50 mM MES pH 6.6, 5% glycerol, 8 mM CaCl_2_, 1.5 M NaCl. In a final polishing step, the NA was fractionated on a Superdex200 pg column, equilibrated with 50 mM MES pH 6.6, 5% glycerol, 8 mM CaCl_2_, 150 mM NaCl. Purified recombinant N1 and N2 NA were aliquoted and stored at −80 °C before use.

The neuraminidase inhibition assay was adapted from the WHO protocol [30]. Shortly, 16 ng of N1 or N2 were incubated overnight with the various concentration of RpSP-D, Concanavalin A (positive control, [31]) or peanut agglutinin (negative control) (Sigma Aldrich Chemie BV, Germany, Steinheim) and 25 µl of fetuin in micronic tubes. Volume was adjusted to 75 uL with PBS-CM. On the next day, 5 µl of periodate reagent (4.28 g of sodium meta-Periodate in 38 ml H_2_O) (Merck-Schuchardt, Germany, Hohenbrunn) was added and the mixture was incubated for 15 min at room temperature. Then 25 µl of arsenite reagent (10 g sodium arsenite, 7.1 g anhydrous sodium sulfate and 100 ml H_2_O) (E Merck, Germany, Darmstadt) was added and mixed to the rest until the brown color disappeared. The mixtures were transferred to a PCR plate. Then 50 µl of thiobarbituric acid reagent (35.5 g anhydrous sodium sulfate, 3.0 g thiobarbituric acid and 500 ml H_2_O) (Sigma Aldrich Chemie BV, Germany, Steinheim) was added and incubated for 15 min at 99°C and 30 min on ice. The mixtures were transferred to micronic tubes, 200 µl of Warrenhoff reagent was added and each tube was vortexed. Finally, the tubes were centrifuged for 5 min at 2000 rpm and 100 µl of the upper butanol phase was transferred to an ELISA plate to measure the optical density with a spectrophotometer (Infinite M200, Tecan) at wavelength 549 nm. The percentage of inhibition was calculated according to the formula, (1-(Absorbance (sample)/Absorbance (without inhibitor)) x100%.

## Results

### Biological activity of RpSP-D resembled that of NpSP-D

**Figure 1:**
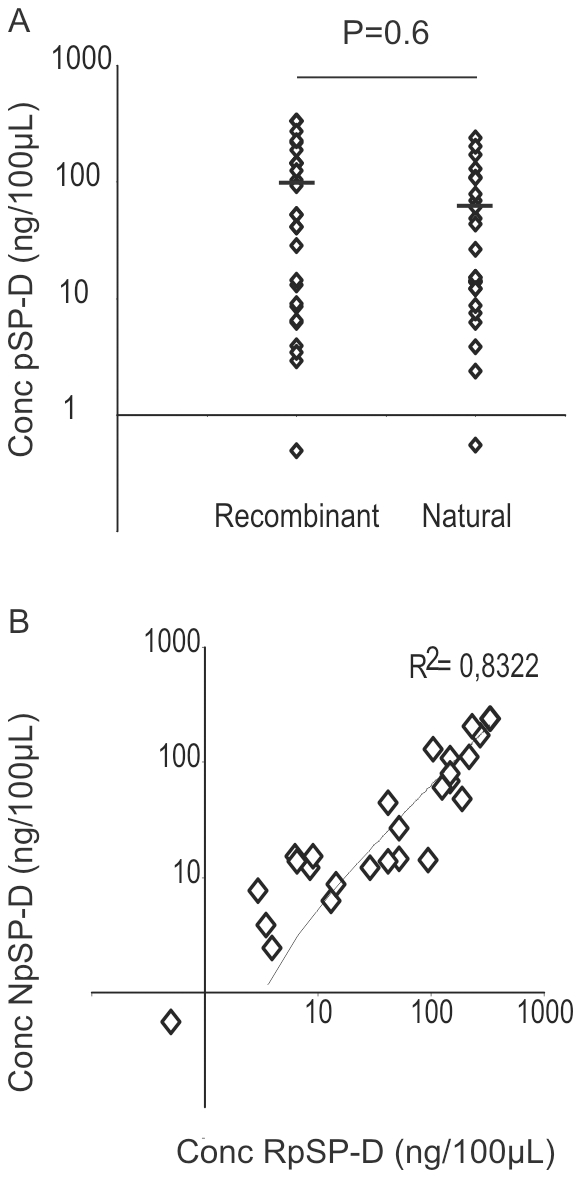
Antiviral activity of RpSP-D resembles that of NpSP-D. The minimal inhibitory concentration of RpSP-D and NpSP-D required to inhibit the hemagglutination by all 27 H1N1 and H3N2 IAVs was assessed (A). The horizontal line indicates the average of the minimal inhibitory concentration for both preparations which did not differ significantly. The values were also compared directly (B). The minimal inhibition concentrations of RpSP-D correlated well with those of NpSP-D. Each symbol in panels A and B represent an individual IAV strain.

First, we investigated whether RpSP-D had similar biological activity against IAV compared to NpSP-D isolated from pig lungs. To this end, the two preparations were tested against 15 H1N1 and 12 H3N2 IAV of swine, human and avian origin. HI assays were performed three times independently and the results are shown in figure 1. RpSP-D inhibited hemagglutination by IAV to a similar extent as NpSP-D and statistical differences were not observed. Furthermore, the activity of both preparations was shown to be dependent on the presence of calcium ions, since no inhibition was observed in PBS without CaCl_2_. In this assay, peanut agglutinin, which was included as a control lectin, did not inhibit IAV-mediated hemagglutination.

### Influenza A viruses of different subtypes are recognized differentially by RpSP-D

**Figure 2:**
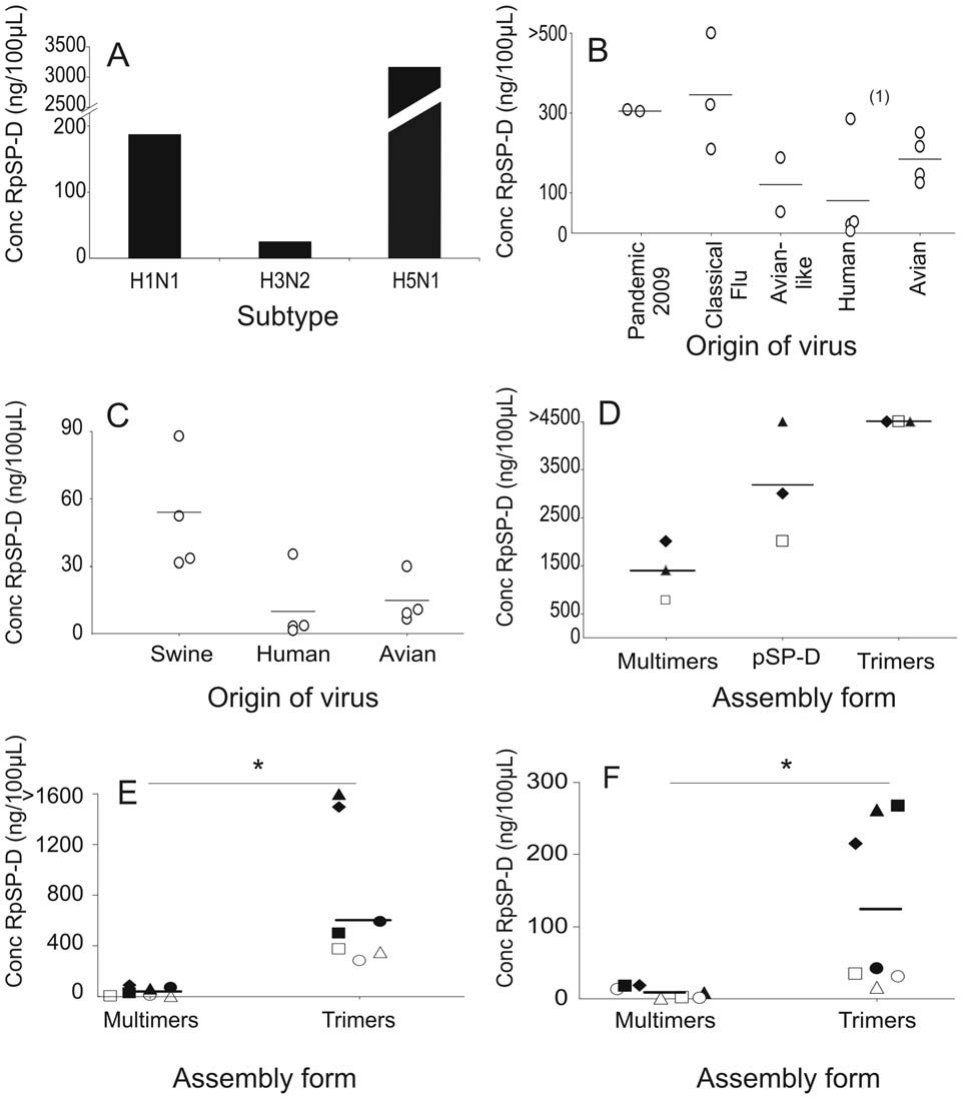
RpSP-D inhibits the hemagglutination by a broad range of IAVs. The minimal inhibitory concentration of RpSP-D that still inhibited the hemagglutination by a variety of IAV was determined. In panel A, the bars represent the average minimal concentration of RpSP-D required to inhibit all H1N1, H3N2 and H5N1 viruses tested, independent of the animal species they originated from. Panels B and C show the results of individual H1N1 (B) and H3N2 (C) viruses and their origin. The average concentrations obtained in six independent experiments are shown. The results obtained in four independent experiments with H5N1 viruses, VietNam/04 (open square), Indonesia/05 (closed diamond), HongKong/97 (closed triangle) are shown in panel D. Also the results (the average of three independent experiments) are shown in panel E and F for inhibition by multimers and trimers of RpSP-D. The H1N1 viruses tested are NL/09 (closed diamond), S/NL/1/87 (closed triangle), NJ/76 (closed square), NL/06 (open square), M/NL/06 (closed circle), NL/08 (open circle), WGF/NL/07 (open triangle) (panel E) and the H3N2 viruses tested are S/NL/93 (closed diamond), NL/07 (open square), NL/03 (open triangle), NL/93 (closed triangle), M/NL//08 (open circle), M/Sweden/03 (closed circle), M/NL/07 (closed square) (panel F).

The minimal concentration of RpSP-D that still prevented hemagglutination by each of the viruses tested was determined. Each assay was performed in duplicate and repeated three to six times and the average minimal inhibitory concentration of RpSP-D was calculated for each virus and plotted in figure 2A–D.

On average, less RpSP-D was required to prevent hemagglutination by IAV (swine human and avian) of the H3N2 subtype (25 ng/100 µl) than those of the H1N1 (187 ng/100 µl) or H5N1 (3167 ng/100 µl) subtype (figure 2A).

Within the H1N1 and H3N2 subtypes, differences in sensitivity for the inhibitory activity of RpSP-D existed, depending on the species the viruses originated from. Apart from PuertoRico/8/34, human IAVs of the H1N1 subtype were susceptible to the inhibitory activity of RpSP-D. On average, 17 ng/100 µl was already sufficient to fully inhibit hemagglutination against the human H1N1 IAVs (excluding PuertoRico/8/34). For avian, avian-like swine and classical swine viruses of the H1N1 subtype these concentrations were higher, 183 ng/100 µl, 120 ng/100 µl and 342 ng/100 µl respectively (figure 2B). The classical swine virus Swine/Iowa/15/30 was not susceptible to inhibition by RpSP-D at the maximum concentration tested (>500 ng/100 µl). Also the 2009 pandemic H1N1 strains, California/4/09 and Netherlands/602/09 were relatively susceptible; the average inhibitory concentration was 304 ng/100 µl (figure 2B).

Also within the H3N2 subtypes, the human-derived strains were more susceptible than the avian and swine viruses with minimal inhibitory concentrations of 11 ng/100 µl, 14.6 ng/100 µl and 51 ng/100 µl respectively (figure 2C).

All viruses of the H5N1 subtype tested, HongKong/156/97 (clade0), VietNam/1194/04 (clade1) and Indonesia/5/05 (clade2.1) were not in particular susceptible and high concentrations of RpSP-D were required to prevent the hemagglutination by these viruses (4500 ng/100 µl, 2000 ng/100 µl and 3000 ng/100 µl respectively) (figure 2D).

### RpSP-D multimers inhibit IAV more efficiently than trimers

HEK293 cell-derived RpSP-D and RhSP-D preparations were assembled in two forms: partly assembled trimeric subunits and fully-assembled oligomeric forms of trimers referred to as multimers. After separation of these two forms by gel filtration chromatography, the inhibitory capacity of both forms was compared with the respective viruses in the HI assay.

As shown in figure 2D, E and F, the specific biological activity of the multimers was greater than that of the trimers. On average, 39.8 ng/100 µl of RpSP-D multimers was required to inhibit hemagglutination by all influenza A/H1N1 viruses tested, which was significantly lower (p = 0.001, Mann-Whitney test) than the minimal inhibitory concentration of the trimers (601.5 ng/100 µl). Influenza virus A/swine/Netherlands/1/87 was not inhibited by the trimers of RpSP-D at the minimal concentration tested (1600 ng/100 µl) (figure 2E). Also for the inhibition of all the influenza A/H3N2 viruses tested the minimal inhibitory concentration of multimers was significantly lower (p = 0.002, Mann-Whitney test) than that of the trimers (8.7 ng/100 µl 124 ng/100 µl respectively) (figure 2F).

The minimal inhibitory concentration of multimers required to inhibit the H5N1 viruses was 1400 ng/100 µl whereas the trimers of RpSP-D failed to inhibit these viruses at the maximum concentration tested (>4500 ng/100 µl) (Figure 2D).

### Comparison of RpSP-D and RhSP-D

Although RpSP-D and RhSP-D have similar structures, there are also some striking differences. Therefore we compared the inhibitory capacity of SP-D derived from these two species in the HI assay using a panel of altogether 27 of H1N1 and H3N2 IAVs.

**Figure 3:**
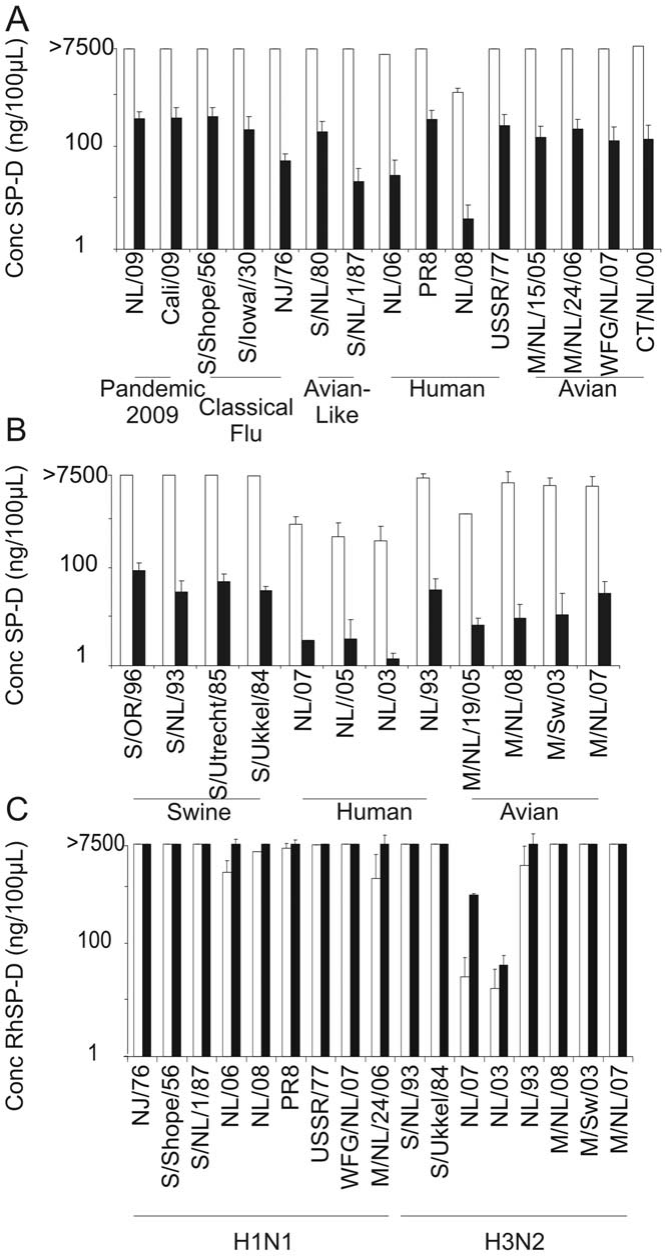
Comparison between the HI activity of RpSP-D and that of RhSP-D. The minimal inhibitory concentrations of RpSP-D (black bars) and RhSP-D (white bars) were determined with the HI assay for the H1N1 (A) and H3N2 (B) viruses as indicated. In panel C, the minimal inhibitory concentrations of RhSP-D multimers (black bars) and trimers (white bars) were compared. The data represent the average of three independent experiments.

It was found that RpSP-D inhibited IAV of the H1N1 subtype more efficiently than RhSP-D. RhSP-D failed to inhibit the hemagglutination by most H1N1 viruses at the maximum concentration tested (>7500 ng/100 µl) (figure 3A). The minimal inhibitory concentration of RpSP-D ranged from 4 ng/100 µl to 318 ng/100 µl.

A similar pattern was observed for the inhibition of the all A/H3N2 viruses tested of which some were inhibited by RhSP-D, albeit at relatively high concentrations ranging from 427 ng/100 µl to 6666 ng/100 µl (figure 3B). In comparison, RpSP-D inhibited hemagglutination by H3N2 efficiently. For all H1N1 and H3N2 viruses, the differences in minimal inhibitory concentration between RpSP-D and RhSP-D were statistically significant (p<0.05). In all cases where inhibition was observed by RhSP-D or RpSP-D, the action of SP-D was dependent on the presence of calcium ions (data not shown). To test whether multimers of RhSP-D would be more potent than the trimeric RhSP-D forms, the inhibitory capacity of RhSP-D multimers was compared to that of the trimers (figure 3C) using a selection of H1N1 and H3N2 IAV (figure 3C). Apart from two individual strains (Netherlands/312/03 and Netherlands/348/07), the minimal inhibitory concentration of multimers was only slightly lower than that of trimers, indicating that the modest inhibitory capacity of RhSP-D was not caused by absence of multimers in the RhSP-D preparation.

### RpSP-D binds to HA

**Figure 4:**
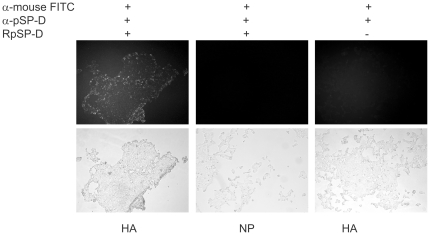
RpSP-D binds to HA. 293T cells were transfected with plasmids expressing HA or NP genes derived from influenza virus A/Netherlands//178/95 (H3N2). The cells were then incubated with RpSP-D or not and binding of RpSP-D was assessed by an immuno-fluorescence assay (upper panels) using a mouse monoclonal anti-pSP-D antibody and a goat anti-mouse FITC labeled antibody. Cells were analyzed using a fluorescence microscope. No binding of RpSP-D was detected in cells transfected with a plasmid encoding the NP gene, which was included as negative control, whereas cells expressing the HA gene bound RpSP-D.

Using immuno-fluorescence, we demonstrated that RpSP-D can bind to HA of human influenza viruses. 293T cells were transfected with plasmids from which the HA and NP genes of influenza virus A/Netherlands/178/95 (H3N2) were expressed. Using a SP-D specific monoclonal antibody, binding of RpSp-D was observed to cells transfected with the plasmid expressing the HA gene but not with cells transfected with a plasmid encoding the NP gene (Figure 4). Without RpSP-D, no fluorescence was observed confirming the specificity of the staining. With plasmids encoding the HA and NP genes of influenza virus A/Netherlands/26/07 (H1N1) similar results were obtained (data not shown).

### RpSP-D inhibits MDCK cells infection with IAV

**Figure 5:**
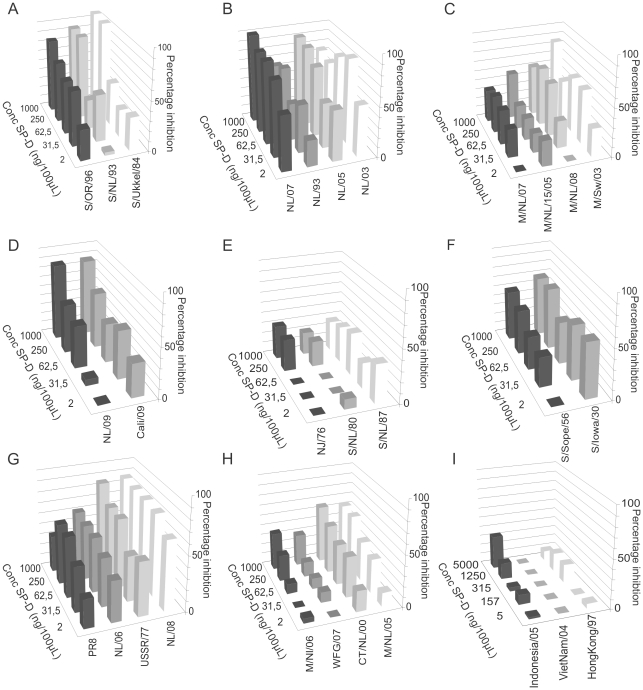
Reduction of IAV infection by RpSP-D. Using the infection reduction assay, the neutralization of IAV by various doses of RpSP-D was assessed. The reduction of infectivity was expressed as the relative number of cells that became infected according to the formula: % reduction = 1-(% infected cells in presence of RpSP-D/% infected cells without RpSP-D)*100%. The viruses were grouped by subtype and origin: swine H3N2 (A), human H3N2 (B), avian H3N2 (C), 2009 pandemic H1N1 (D), avian-like swine H1N1 (E), classical swine (F), human H1N1 (G), avian H1N1 (H) and H5N1 (I). The average of triplicate wells is shown.

Using an infection reduction assay, the capacity of RpSP-D to protect MDCK cells from infection with the respective viruses was tested. Peanut agglutinin, which was included as a negative control, failed to reduce the number of cells infected with any of the IAVs used (data not shown). In contrast, RpSP-D inhibited infection of MDCK cells by a wide range of viruses of the H1N1, H3N2 and H5N1 subtype (Figure 5). For most IAV strains tested, a dose-dependent reduction of the number of infected cell was observed.

In this cellular infectivity assay, none of the viruses tested were fully inhibited by SP-D. At the highest concentration tested (1000 ng/200 µl), on average 77.7% inhibition was observed for the pandemic 2009 H1N1 viruses, 47.2% for the classical swine viruses, 24.9% for the avian-like swine viruses and 68.5% for the human seasonal H1N1 viruses (except A/PuertoRico/8/34).

Viruses of the H3N2 subtype were inhibited more efficiently and at the highest dose of RpSP-D tested, 84.9% inhibition was achieved for the swine H3N2 viruses, 72.9% for the human and 52.3% for the avian H3N2 viruses. At the lowest concentration tested (2 ng/200 µl) a reduction of infection of 45.6% was observed with the human H3N2 viruses and 29.6% with the human H1N1 viruses.

The viruses of the H5N1 subtype were poorly inhibited even when a high concentration of RpSP-D was used (5000 ng/200 µl). IAV strain VietNam/1194/04 was not inhibited at all while for HongKong/156/97 and Indonesia/5/05 only 10% and 34% inhibition was observed, respectively.

### RpSP-D inhibits the enzymatic activity of the viral neuraminidase

We also determined whether RpSP-D can interact with NA and is able to inhibit its enzymatic activity. To this end, purified recombinant neuraminidase of the N1 and N2 subtype instead of virion-exposed NA was used to exclude possible steric hindrance of the binding of RpSP-D to HA.

**Figure 6:**
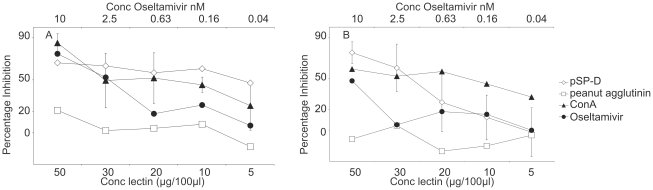
Inhibition of neuraminidase activity by RpSP-D. The enzymatic activity of recombinant purified neuraminidase derived from IAV strains A/crested eagle/Belgium/01/2004 (N1) (A) and A/Victoria/3/75 (N2) (B) was determined after overnight incubation in the presence of various doses of peanut agglutinin (open square), ConA (open circle), oseltamivir (closed circle) or RpSP-D (closed triangle). The concentration is expressed in μg/100μl for the lectins and in nM for oseltamivir. NA activity in the absence of inhibitors was normalized to 100% and the percentage of inhibition was depicted.

As shown in figure 6, peanut agglutinin did not inhibit the enzymatic activity of neuraminidase significantly.

In contrast, both RpSP-D and ConA inhibited enzymatic activity of both subtypes of NA tested, although relatively high doses of RpSP-D were required to achieve maximal inhibition.

A concentration of 50 µg/100 µl inhibited the enzymatic activity the NA of A/crested eagle/Belgium/01/2004 (H5N1) by 85% and the activity of A/Victoria/3/75(H3N2) by 75%. As expected, oseltamivir inhibited neuraminidase activity efficiently (figure 6). In the assay used, oseltamivir inhibited 75% of the enzymatic activity the N1 molecule and 48% of the N2 molecule at a concentration of 10 nM.

### RpSP-D inhibits binding of virus to ciliated epithelial cells of ferret and human trachea

To be effective as an antiviral drug, RpSP-D must be able to interfere with binding of virus to cells of the respiratory tract. Therefore, we tested the capacity of RpSP-D to inhibit attachment of a human seasonal H1N1 and a human seasonal H3N2 virus to tracheal epithelial virus cells using virushistochemistry.

**Figure 7:**
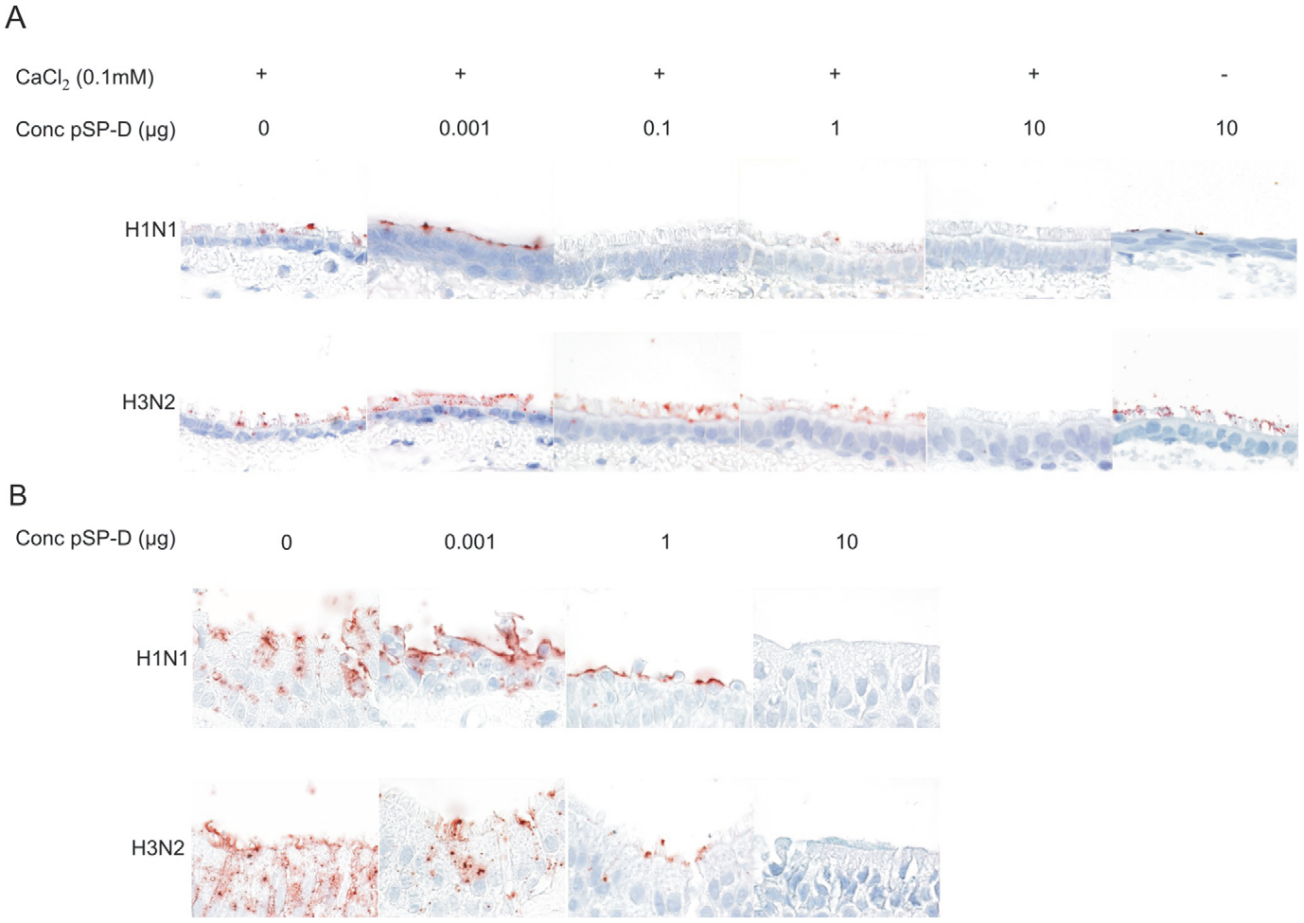
RpSP-D prevents binding of human seasonal H1N and H3N2 viruses to epithelial cells of ferret and human trachea. Attachment of virus in absence or presence of RpSP-D was studied by virushistochemistry. Sections of ferret trachea (A) or human trachea (B) were incubated with FITC-labeled IAV A/Netherlands/35/05 (H1N1) and A/Netherlands/231/03 (H3N2) in the absence or presence of various doses of RpSP-D as indicated. Binding of virus is visible as dark-red staining. As a negative control, the highest dose of RpSP-D (10 µg) was also measured in absence of calcium ions. The tissues were counterstained with hematoxylin (magnification x100).

As shown in figure 7A, both viruses bind to epithelial cells of ferret trachea which is visible as red precipitate. Preincubation of FITC-labeled virus with RpSP-D prevented binding at a minimal dose of 0.1 µg for the H1N1 virus and 10 µg for the H3N2 virus. A dose of 1 µg did not prevent the binding of the H3N2 virus completely but the number of positive cells was reduced compared to that after incubation of labeled virus in the absence of RpSP-D. The inhibition of virus attachment was dependent on the presence of Ca^2+^-ions, since in the absence of CaCl_2_, a dose of 10 µg RpSP-D failed to prevent attachment of both viruses.

Two control lectins, peanut agglutinin and pokeweed mitogen, failed to prevent virus attachment. In contrast, ConA, which was included as a positive control since it has high affinity for the viral hemagglutinin, blocked virus attachment at a concentration of 1 µg/150 µl (data not shown).

We also tested the effect of RpSP-D on virus attachment using human trachea tissue (figure 7B). At a dose of 10 µg, RpSP-D fully prevented binding of human seasonal H1N1 and H3N2 IAV. At a dose of 1 µg, binding of virus to epithelial cells was reduced considerably compared to virus attachment in the absence of RpSP-D.

## Discussion

In the present study, the antiviral activity of RpSP-D was assessed against a wide range of IAV strains.

Collectins are C-type lectins and function as antimicrobial defense molecules of the innate immune system [14]. For the development of a collectin-based antiviral drug, the use of a well-defined recombinant product is the most acceptable venue. However, the biological properties of the recombinant protein need to resemble those of the native protein. First, the biochemical properties of RpSP-D were characterized in detail and compared with NpSP-D and it was shown that RpSP-D is structurally and functionally identical to NpSP-D (van Eijk et al., manuscript in preparation). In this study, we focused on the antiviral properties of RpSP-D and compared its IAV-neutralizing activity with that of NpSP-D isolated from pig lungs in the HI assay against a broad panel of IAV strains. The inhibitory activity of both preparations was comparable and dependent on the presence of calcium ions, indicating that we were able to produce a biological active and properly folded recombinant protein.

We also compared the antiviral activity of RpSP-D with that of RhSP-D. In general, RpSP-D had a more potent antiviral activity than RhSP-D as measured in the HI assay. For example, the two 2009 H1N1 pandemic strains were not susceptible to inhibition by RhSP-D, which is in agreement with a previous study [32]. In contrast, RpSP-D did inhibit hemagglutination by 2009 H1N1 viruses although relatively high doses were required. Furthermore, RhSP-D failed to inhibit the hemagglutination by swine IAV of the H1N1 subtype. Avian H3N2 and human H3N2/H1N1 viruses were inhibited inefficiently since at least 100-fold more RhSP-D than RpSP-D was required.

Thus it was concluded that RpSP-D inhibited a broader range of IAVs and more effectively than RhSP-D and was therefore studied in more detail.

RpSP-D not only inhibited hemagglutination by most H1N1 and H3N2 viruses, it also reduced infection of MDCK cells by these viruses. Only viruses of the H5N1 subtype were inhibited inefficiently and very high doses were required to observe inhibition in both assays. RhSP-D also failed to neutralize viruses of this subtype as demonstrated previously [33].

It is of interest to note that human H1N1 and H3N2 viruses were more susceptible to the action of RpSP-D and RhSP-D than those originating from pigs and birds species.

These differences may be explained by differences in glycosylation. The HA of human IAV contains more putative N-linked glycosylation sites than avian and swine viruses allowing SP-D to interact with the HA more efficiently through its CRD domain as was shown for RhSP-D [32], [33], [34].

The potency of RpSP-D was superior to that of RhSP-D, which may be explained by structural differences. Compared to RhSP-D, RpSP-D has an additional loop in its CRD, an additional glycosylation site and an additional cysteine in the collagen domain. It has been shown that the sialic acid-rich N-linked glycan in the CRD provides an additional mode of interaction, most likely with the sialic acid receptor present at the tip of the viral hemagglutinin molecule [20]. However the contribution of each of these features to the superior antiviral activity of RpSP-D needs to be further elucidated.

The observation that fully assembled RpSP-D neutralizes IAV better than the trimeric form is in line with the reasoning mentioned above. Most likely multimerisation increases the avidity of the SP-D molecules, by increasing the number of CRDs that can interact with IAV simultaneously.

Another potential viral target that could be bound or inhibited by RpSP-D is the NA of IAV. It has been shown previously that inhibition of the enzymatic activity of NA successfully inhibited virus replication and viral spread and a class of existing antiviral drugs is based on this principle. To investigate whether IAV NA, which is also glycosylated, can be inhibited by RpSP-D, we used, in contrast to other studies [33], [35], recombinant NA molecules to exclude the interference of the enzymatic activity by binding of RpSP-D to HA. Although the enzymatic activity was inhibited to a certain extent, relatively high RpSP-D doses were required to achieve this. We therefore conclude that binding of RpSP-D to the viral HA most likely is a more potent mode of antiviral action than binding to NA. Of note, the reduction of infection of MDCK cells that was observed correlated with the inhibition of the hemagglutination activity of IAV. This provides additional evidence that HA is the primary target of RpSP-D. The interaction of RpSP-D with the viral HA, prevents the attachment of the virus to target cells and is at the basis of the neutralizing effect of RpSP-D. Indeed, we demonstrated that RpSP-D is able of binding HA of influenza A viruses of the H1N1 and H3N2 subtype (Figure 4).

Using tissue sections of ferret and human trachea and virushistochemistry we demonstrated that RpSP-D could reduce binding of human IAV H1N1 and H3N2 viruses to epithelial cells of the human and ferret upper respiratory tract. The binding of virus to paraffin-embedded tissues may not reflect the virus-tissue interaction *in vivo* exactly. However, the binding patterns of various influenza viruses to respiratory tract tissue obtained from various animal species correlate with pathogenesis and transmissibility of these viruses. This indicates that the patterns of virus-tissue binding assessed by our virushistochemistry method has biological relevance [27], [36], [37], [38].

In conclusion, the results obtained in the present study showed that RpSP-D has potent antiviral activity against a wide range of IAV strains *in vitro*. RpSP-D had broad neutralizing activity against IAVs which were neutralized differentially, depending on subtype and animal species of origin. The antiviral effect is mostly mediated by binding of RpSP-D to the viral HA, thereby preventing it from binding to the host cells receptors as was demonstrated by virushistochemistry using ferret and human trachea tissue. Future studies will aim at the delivery of RpSP-D and assessing its antiviral properties *in vivo*.
